# Normal temperature upon admission does not influence on timing of antibiotics for septic patients

**DOI:** 10.1186/1757-7241-21-S2-A7

**Published:** 2013-09-09

**Authors:** Anne-Katrine Bertelsen, Julie Mackenhauer, Nina Buch, Helle Nibro, Hans Kirkegaard

**Affiliations:** 1 Research Center for Emergency Medicine, Aarhus University Hospital, Denmark; 2 Intensiv Terapi Afsnit (ITA), Aarhus University Hospital, Denmark; 3 The CONSIDER sepsis network, Research Center for Emergency Medicine, Aarhus, Denmark

## Background

Early identification and treatment of sepsis is essential for prognosis and outcome. Sepsis is a complex syndrome based on non-specific symptoms, making early identification a medical challenge. Elevated or lowered bodytemperature often release a blood culture. Our hypothesis is, that a lack of temperature upon admission influence on the time of diagnosis and thereby time of antibiotics.

## Methods

Our cohort is a part of a larger clinical database of septic patients identified through a prospective screening of all patients admitted to the intensive care unit (ICU) at Aarhus University Hospital from Nov 2008 - Sep 2010. Patients above age 18 admitted directly from the Emergency Department (ED) to the ICU with severe sepsis or septic shock were included. We compared patients with elevated (≥38°C) or lowered (≤36°C) bodytemperature to patients who demonstrated a normal temperature upon admission to the ICU, relative to initiating empirical antibiotic treatment.

## Results

A total of 180 septic patients were admitted to the ICU directly from the ED.

161 had a temperature registered upon arrival to the ICU. 52% had an abnormal temperature.

There were no difference regarding age and gender between the two groups. Overall in-hospital mortality was 21,1%.

Comparing patients with abnormal temperature to patiens with normal temperature upon admisssion, we found no difference in timing of antibiotics. 53% of the patients with abnormal temperature recieved antibiotics in the ED, while 47% of the patients with normale temperature recieved antibiotics prior to admission to the ICU (p=0,62), The remainding recieved antibiotics in the ICU.

Comparing our findings to local guidelines for ”timely antibiotics”, 67% with abnormal and 64% with normal temperature recieved antibiotics according to the guidelines.

## Conclusion

In a population of septic patients admitted directly from the ED to the ICU, we found no difference in timing of antibiotics between patients with normal temperature and patients with abnormal temperature upon admission. Increasing focus on sepsis, and use of other clinical indicators of infection may contribute to our findings. Only 2/3 of the population recieved timely antibiotics accoring to local guidelines for sepsis treatment.

